# Nitrite-oxidizing bacteria adapted to low-oxygen conditions dominate nitrite oxidation in marine oxygen minimum zones

**DOI:** 10.1093/ismejo/wrae160

**Published:** 2024-08-14

**Authors:** Samantha G Fortin, Xin Sun, Amal Jayakumar, Bess B Ward

**Affiliations:** Department of Geosciences, Princeton University, Princeton, NJ 08544, United States; Department of Global Ecology, Carnegie Institution for Science, Stanford, CA 94305, United States; Department of Geosciences, Princeton University, Princeton, NJ 08544, United States; Department of Geosciences, Princeton University, Princeton, NJ 08544, United States

**Keywords:** nitrite-oxidizing bacteria, oxygen minimum zone, nitrification

## Abstract

Nitrite is a central molecule in the nitrogen cycle because nitrite oxidation to nitrate (an aerobic process) retains fixed nitrogen in a system and its reduction to dinitrogen gas (anaerobic) reduces the fixed nitrogen inventory. Despite its acknowledged requirement for oxygen, nitrite oxidation is observed in oxygen-depleted layers of the ocean’s oxygen minimum zones (OMZs), challenging the current understanding of OMZ nitrogen cycling. Previous attempts to determine whether nitrite-oxidizing bacteria in the anoxic layer differ from known nitrite oxidizers in the open ocean were limited by cultivation difficulties and sequencing depth. Here, we construct 31 draft genomes of nitrite-oxidizing bacteria from global OMZs. The distribution of nitrite oxidation rates, abundance and expression of nitrite oxidoreductase genes, and relative abundance of nitrite-oxidizing bacterial draft genomes from the same samples all show peaks in the core of the oxygen-depleted zone (ODZ) and are all highly correlated in depth profiles within the major ocean oxygen minimum zones. The ODZ nitrite oxidizers are not found in the Tara Oceans global dataset (the most complete oxic ocean dataset), and the major nitrite oxidizers found in the oxygenated ocean do not occur in ODZ waters. A pangenomic analysis shows the ODZ nitrite oxidizers have distinct gene clusters compared to oxic nitrite oxidizers and are microaerophilic. These findings all indicate the existence of nitrite oxidizers whose niche is oxygen-deficient seawater. Thus, specialist nitrite-oxidizing bacteria are responsible for fixed nitrogen retention in marine oxygen minimum zones, with implications for control of the ocean’s fixed nitrogen inventory.

## Introduction

Nitrite is produced and consumed by microbial processes, including denitrification, nitrate respiration, anammox, and nitrification, which require either presence or absence of oxygen. Oceanic oxygen minimum zones (OMZs) are characterized by a permanent subsurface oxygen-deficient zone (ODZ) and a corresponding secondary nitrite peak. OMZs are important hotspots of nitrogen cycling leading to either the conservation, driven by nitrite oxidation to nitrate, or loss, driven by nitrite reduction to dinitrogen gas during denitrification and anammox, of fixed nitrogen. These two steps are assumed to be independent because nitrite oxidation is understood to be an obligately aerobic process, and denitrification and anammox are anaerobic. Evidence [1–4] that nitrite oxidation can occur in samples collected from oxygen-depleted seawater, i.e. seawater where oxygen is below the detection limit and denitrification and anammox occur, therefore places nitrite firmly at the pivot point—the fate of nitrite determines the rate of fixed nitrogen loss. Because the inventory of fixed nitrogen controls primary production across much of the world’s oceans [5], the balance of nitrogen retention and removal in OMZs can have significant impacts on ocean biogeochemistry.

In OMZs, nitrite oxidation rates consistently exceed rates of ammonia oxidation, the first step in nitrification, and highest rates often occur in the absence of detectable oxygen [1–4, 6–11]. Both anammox bacteria and nitrite-oxidizing bacteria (NOB) oxidize nitrite to nitrate using the enzyme nitrite oxidoreductase (NXR) [12, 13]. The depth distribution of measured rates of anammox (i.e. N_2_ production from NH_4_^+^ or NO_2_^−^) is quite different from the distribution of total nitrite oxidation, and the stoichiometry of anammox means that its associated rates of nitrate production are very low. Therefore, most of the observed nitrite oxidation in OMZs cannot be attributed to anammox [6].

Nitrite oxidation, anammox, and denitrification have all been quantified in isotope tracer experiments using natural seawater samples in the same incubation bottles [4, 14], challenging the previous view of the spatial separation of N retention and loss steps. Nitrite oxidation and nitrate reduction rates in oxygen-depleted waters are highly correlated, suggesting an additional nitrogen loop centered in oxygen-depleted waters [4, 15], in which nitrite is rapidly recycled to nitrate at much greater rates than net reduction to dinitrogen gas by anammox or denitrification. Natural abundance isotope data and modeling [16, 17] argue that observed natural abundance isotopic composition of nitrite and nitrate in oxygen-depleted waters cannot be explained without nitrite oxidation. The stoichiometry of nutrient remineralization indicates that most of the nitrite produced in OMZs is reoxidized *in situ* [18], which substantially decreases the loss of fixed nitrogen in OMZs [14] and increases the retention of fixed nitrogen in the open ocean.

*Nitrospinae* are the most abundant NOB in the ocean and contribute to dark carbon fixation in deep, oxygenated waters [19]. Autotrophic carbon fixation, even using the efficient reductive TCA (rTCA) pathway conserved across all known *Nitrospinota* [20], requires the oxidation of large amounts of nitrite, which explains the finding that NXR enzymes affiliated with *Nitrospinae* are among the most abundant proteins in the ocean [21, 22]. NOB belonging to *Nitrospinae* Clade 1a and Clade 2 and *Nitrospirae* exhibited vertical, but not latitudinal, biogeography driven by differential adaptations to oxygen and nutrient conditions [19]. In OMZs, *Nitrospina* are the most common NOB, based on transcription of *nxr* [23], and nitrite oxidation rates in the Eastern Tropical South Pacific (ETSP) are correlated with *Nitrospina* 16S rRNA gene abundance [24].

Single-amplified genomes (SAGs) and metagenome-assembled genomes (MAGs) of NOB from globally distributed samples show no evidence of heterotrophy in marine *Nitrospinota* [19, 20]. A recent study comparing all known *Nitrospinota* from marine and freshwater sources illuminated the great diversity in the phylum and discovered genetic potential for the use of sulfide and hydrogen in some classes [20]. The particular *Nitrospinia* clades that are abundant in the ocean have not been cultured; their cultivated relatives are obligate aerobes and autotrophs [25], although lack of oxygen defense mechanisms and use of the rTCA cycle suggest microaerophilic ancestry.

The discovery of two Clade 1 *Nitrospina*-like NOB MAGs retrieved from the ETSP OMZ provided evidence that uncultured NOB may be responsible for nitrite oxidation in oxygen-depleted seawater. These MAGs (ETSP2013_BB2_MAG1 and ETSP2013_BB2_MAG2) reached relative abundances as high as 3% in the ODZ [26] and were found in published OMZ metatranscriptomes [23, 27], indicating activity and expression in the ODZs of the Eastern Tropical North Pacific (ETNP) and ETSP [14].

Our understanding of nitrite oxidation in OMZs, and its prevalence in oxygen-depleted waters, is hindered by our lack of understanding of the NOB responsible for this process in these regions. Here, we provide a comprehensive analysis of NOB communities in OMZs by identifying diverse NOB draft genomes (MAGs), exploring their metabolic repertoire, determining their distribution in OMZs and the global ocean, and quantifying the expression of *nxrB* genes. When combined with published nitrite oxidation rates obtained from the same samples, these multiple lines of evidence reveal specialist NOB that dominate oxygen-depleted waters and are responsible for high rates of nitrite oxidation.

## Materials and methods

### Metagenomic sequencing

Deep metagenomic sequencing was performed by the Joint Genome Institute (JGI) on 19 samples, 15 from depth profiles in the ETNP (Stations PS6 and 14 from 2016 and Stations PS2 and PS3 from 2018) and 4 from the Arabian Sea (Station 2 from 2007) (Tables S1 and S6), using a NovaSeq (Illumina). Analysis of the denitrifying community in a subset of these samples was recently reported [28]. Four of these ETNP stations (2016 station PS6, 2018 stations PS1, PS2, PS3) have previously published nitrite oxidation rates from the same exact samples; full details of these rate measurements can be found in [3, 15]. Full details of the cruises and sample collection were published previously [3, 14, 29]. Eight additional metagenomic samples (Table S1) were collected from the ETNP in 2018 at Stations PS2 and PS3 for a concurrent, currently unpublished, study. These additional eight samples were used only for binning MAGs; nitrite oxidation was not measured, and these samples are not included in the depth profiles. Details of the collection and processing of these samples can be found in supplemental methods. All metagenomic samples were collected on Sterivex (0.22 μm) filters, frozen immediately in liquid N_2_ before being stored at −80°C, and extracted using the All-Prep DNA/RNA Mini Kit (Qiagen, Valencia, CA) and manufacturer’s protocols.

### Bioinformatics

Bioinformatic analyses were performed on a combination of the KBase [30] platform and Princeton University’s High-Performance Computing servers. Sequences produced by JGI were quality controlled and trimmed by JGI protocols using BBDuk [31]. The remaining sequences were quality-controlled and trimmed using TrimGalore (https://github.com/FelixKrueger/TrimGalore) with a minimum quality score of 30, a stringency of 3, and a minimum length of 50.

Metagenomic reads from each sample were individually assembled using MEGAHIT v1.2.9 [32] with the meta-sensitive preset and a minimum contig length of 1000 bp. Each assembly was binned into MAGs using three separate binning programs, CONCOCT v1.1 [33], MaxBin2 v2.2.4 [34], and MetaBAT2 v1.7 [35]. Binning programs were set to accept a minimum contig length of 1000 bp except for MetaBAT2 (minimum contig length 1500 bp), and all programs used default settings with the exception that CONCOCT mapping was performed with bowtie2 very-sensitive presets. The resulting MAGs were dereplicated using DAS Tool v1.1.2 [36] to obtain a set of dereplicated MAGs for each sample. The quality of all MAGs was checked with CheckM v1.0.18 [37], and MAGs were taxonomically identified as NOB using GTDB-Tk v2.3.2 [38].

The average nucleotide identity (ANI) of each ODZ NOB MAG obtained from this study, calculated with FastANI [39], was compared to the other ODZ NOB and 61 dereplicated *Nitrospinota* genomes representing the full range of known marine *Nitrospinota* from cultures, MAGs, and SAGs (Table S3) [20]. ANI pairwise comparisons, which calculates ANI on shared regions of the genome and could therefore underestimate the true phylogenetic difference between distantly related organisms, were used to group NOB MAGs into species equivalents with a cutoff of 95% [40]. The best (highest-quality) MAG representing each species equivalent NOB group was chosen based on CheckM completion and contamination results with less contamination favored over a higher completeness for similar MAGs; representative MAGs (Table 1) for each group were substantially (>70%) or nearly (>90%) complete with medium (<10%) or better (<5%) contamination except for NOB6 and NOB8, which were moderately (>50%) complete (Table S2) [37]. These representative MAGs were used to calculate relative abundance for all ODZ NOB. In addition, representatives of *Nitrospinae* Clade 1a (AG-538-K21, called SAG_1a throughout), Clade 2a (AG-538-L21, called SAG_2a throughout), and *Nitrospirae* (AC-732-L14, called SAG_Nitrospirae throughout) are used to represent common NOB from oxygenated marine ecosystems (Table S3).

**Table 1:** Completeness and contamination of each representative ODZ NOB MAG, and the sample from which each MAG was reconstructed.

**NOB Group**	**MAG ID**	**Sample**	**Completeness (%)**	**Contamination (%)**
ODZ NOB1	ODZ_NOB1a	ETNP PS3 70 m TF	96.5	5.2
ODZ NOB2	ODZ_NOB2a	ETNP PS6 200 m	97.2	5.1
ODZ NOB3	ODZ_NOB3a	ETNP PS6 750 m	84.2	3.5
ODZ NOB4	ODZ_NOB4a	ETNP 14185 m	94.6	8.8
ODZ NOB5	ODZ_NOB5a	ETNP PS2 130 m TF	82.0	7.5
ODZ NOB6	ODZ_NOB6a	ETNP PS2 130 m TF	56.6	1.9
ODZ NOB7	ODZ_NOB7a	Arabian Sea 150 m	95.7	1.7
ODZ NOB8	ODZ_NOB8a	Arabian Sea 150 m	67.9	6.7
ODZ NOB9	ODZ_NOB9a	Arabian Sea 200 m	71.9	3.5
ODZ NOB10	ODZ_NOB10a	Arabian Sea 400 m	91.0	1.7

The number of reads in each sample that mapped to each NOB MAG was determined using bowtie2 [41], with very-sensitive presets and end-to-end alignment. All representative NOB MAGs and SAGs, as well as all cultured genomes, were mapped simultaneously, allowing each sample read to be mapped to only one genome. A modified version of RPKM (reads per kilobase per million mapped reads) of each MAG in each sample was then determined by dividing the number of mapped reads by the total number of reads (in million) of the sample and dividing the resulting value by the genome length (kbp) of the MAG [42]. RPKM was used as a measure of relative abundance in order to normalize abundance to both sequencing depth and MAG length. The relative abundance of complete genomes of cultured organisms and previously identified NOB SAGs (Table S3) was determined following the same procedure. The representative NOBs were used to determine the relative abundance of the ODZ NOBs in additional samples from previously published studies (Table S4). NOB communities were compared using principal component analysis (PCoA) performed using phyloseq [43]. Pearson correlation analyses were performed using R [44] (α = 0.05) on samples with a complete set of data, i.e. stations PS6, PS2, and PS3. The relative abundance of cultured organisms was not included in the correlation analysis because many of the species had an RPKM of 0 across all samples.

A pangenome analysis was performed to better understand the genetic makeup of the most abundant and relatively closely related identified NOB, NOB1, NOB2, NOB3, and SAG_1a (AG_538_K21). The *Nitrospina gracilis* genome (strain 3/211) was included in the analysis as a well-characterized, complete genome comparison. The pangenome analysis was performed with anvi’o version 7.1 [45] using the anvi-pan-genome command and default settings; the programs DIAMOND [46] (with sensitive presets), HMMER [47], and prodigal [48] were utilized in the analysis. All ODZ NOB genomes, as well as *N. gracilis*, SAG_1a, and SAG_2a (AG_538_L21) were annotated with DRAM [49]; in addition, anvi’o was used to annotate each genome against the NCBI COGs, KEGG, and pfam databases [50–52]. Annotated genomes were used to search for specific genes of interest.

### Quantification and phylogeny of *nxrB* genes

The beta subunit of the nitrite oxidoreductase gene (*nxrB*) is commonly used for NOB phylogeny and identification [53]; existing primer sets did not capture all the *nxrB* sequences identified from the OMZs so new primers were developed (see supplemental methods). The new primer set, nxrBomz1F (5’ GAGTAYATGTGGTGGAAYAAYGT) and nxrBomz1R (5’ CRTCCTCYTGGCGYTTGTA), was used to detect *nxrB* genes in the OMZ depth profiles with polymerase chain reaction (PCR) and quantitative PCR (qPCR).

SYBR green qPCR was performed to measure the number of *nxrB* gene copies in both DNA and cDNA (created from DNase I-treated RNA) of samples from the ETNP and Arabian Sea OMZs. Double-stranded cDNA was created using the Invitrogen (ThermoFisher, Waltham, MA) SuperScript III First-Strand Synthesis System and the Invitrogen Second-Strand cDNA Synthesis Kit following manufacturer protocols. The *nxrB* standard was diluted serially to make standard curves. Using optimized qPCR assays (see supplemental methods), each sample was run in triplicate and each plate included a negative control; a standard curve was accepted only if the *R*^2^ > 0.99. No nonspecific binding was observed in gel electrophoresis of the amplified samples from PCR or qPCR.

To confirm the identity of NOB actively expressing *nxrB*, cDNA from the depth with the highest rate of nitrite oxidation from Stations PS6, PS1, PS2, and PS3 was cloned (see supplemental methods). Cloned *nxrB* nucleotide sequences were then aligned with *nxrB* sequences from metagenomes, cultured genomes, MAGs, and SAGs using MEGA [54] and their phylogenetic relationship to each other determined using IQ-Tree with default settings, 100 bootstraps, and model selection performed by IQ-Tree (best fit model: TNe + G4) [55]. Trees were visualized using iTOL version 6.6 [56].

## Results

### Nitrite-oxidizing bacteria from oxygen minimum zones

**Figure 1:**
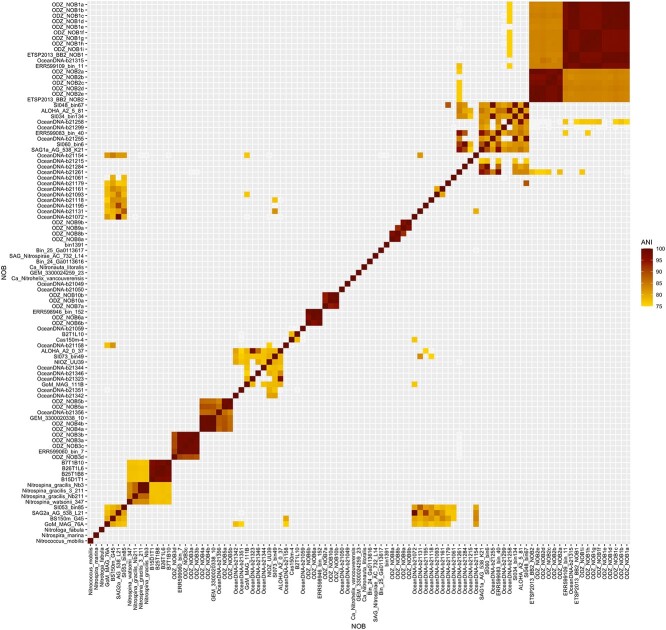
ANI (%) pairwise comparisons between NOB MAGs from this study and the genomes of previously published NOB MAGs, SAGs, and cultured NOB (Table S3). NOB groups were named for the NOB MAG from this study included in the group.

Samples from the ETNP and Arabian Sea OMZs (Table S1, Fig. S1, Fig. S6, supplemental discussion 1) were examined in search of new NOB draft genomes. Thirty-one MAGs, 51.89–97.31% (average 83.11%) completeness with contamination 1.71–14.01% (average 6.74%), were identified taxonomically as NOB (Table S2); 14 contained at least one copy of *nxrB* and 2 MAGs contained 2 copies. These ODZ NOB MAGs separated into 10 distinct groups (or “species,” ANI cutoff 95%; Fig. 1). NOB1 consisted of 5 MAGs from the ETNP and 4 from the Arabian Sea and was the same as MAGs ETSP2013_BB2_NOB1, OceanDNA-b21315, and ERR599109_bin_11 previously binned from the OMZs of the ETSP, ETNP, and Arabian Sea, respectively (Fig. 1; Table S3). NOB2 included 5 NOB MAGs from the ETNP as well as the previously identified ETSP2013_BB2_NOB2. NOB3 included 3 MAGs from the ETNP; a fourth MAG from the Arabian Sea was included in this group despite its ANI of 90%, due to its low completeness (53%), further investigation may place this MAG in a separate group. NOB3 was the same as ERR599060_bin_7 isolated from the ETNP OMZ. The remaining NOB groups included 1 or 2 MAGs from the ENTP and Arabian Sea OMZs with NOB4 grouping with GEM_3300020338_10 from the ETNP OMZ, and NOB6 grouping with MAG ERR598946_bin_152 from the ETSP OMZ (Fig. 1). Each NOB group is represented by a representative MAG (contamination <10%; Table 1) that is used for further analysis.

The ODZ NOB MAGs are only distantly related to cultured NOB and are different from NOB obtained from oxic marine waters and areas with seasonal ODZs like Saanich Inlet and the Gulf of Mexico (Fig. 1; Table S3). Based on GTDB-Tk taxonomy, NOB1, 2, and 3 fall into the class *Nitrospinia*, NOB4 and 5 belong to CAJXCL01 (previously UBA9942), NOB6 belongs to UBA7883, and NOB7, 8, 9, and 10 belong to UBA8248. UBA8248 has recently been re-categorized as its own phylum but was previously placed within *Nitrospinota* so these genomes were retained in the analysis. NOB1, 2, and 3 likely belong to *Nitrospinae* Clade 1, with NOB1 and 2 falling into Clade 1a. *nxrB*-based phylogeny confirms these results: *nxrB* genes from ODZ NOB MAGs were distinct from other *nxrB* genes except for those from *Nitrospinae* Clade 1a which were similar to NOB1 and 2, NOB4 and NOB5 *nxrB* genes group together, and genes isolated from NOB7, 8, and 10 group together on a more distant leaf (Fig. S2).

**Figure 2:**
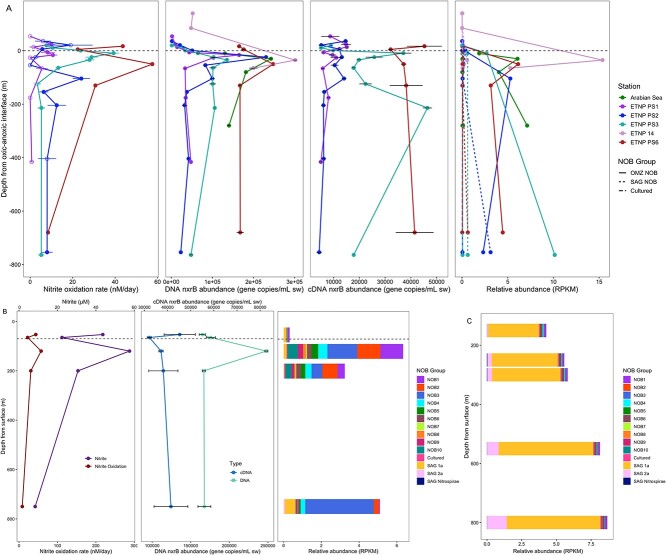
(A) The nitrite oxidation rate (nM/day, previously published in [3, 15]), DNA and cDNA based *nxrB* gene abundance (gene copies/mL seawater), and the relative abundance (RPKM) of NOB groups in each station plotted against the depth above (positive values) or below (negative values) the upper oxic-anoxic interface, defined as the depth where measured O_2_ concentrations fell below 1 μmol/L across all stations (nitrite oxidation rates and cDNA not available for Arabian Sea and ETNP 14; relative abundance not available for ETNP PS1). RPKM shown here was calculated by combining the RPKM of the 10 representative ODZ NOB for the ODZ_NOB group, the 6 cultured organisms for cultured, and the 3 SAGs for SAG_NOB. Error bars represent standard error; nitrite oxidation error bars are from biological replicates, *nxrB* abundance error bars are from technical replicates. (B) The nitrite oxidation rate (nM/day), DNA and cDNA based *nxrB* gene abundance (gene copies/ml seawater), and the relative abundance (RPKM) of NOB in Station ETNP PS6, as an example, plotted against the depth (m) from the surface. (C) The relative abundance (RPKM) of NOB in an ETNP 2011 [21] station at the outer boundary of the OMZ where oxygen concentrations were above 10 μM at all depths; depth is plotted as meters from the surface.

### Abundance and activity of oxygen-deficient zone nitrite-oxidizing bacteria

All 10 ODZ NOB groups were present in all OMZ stations. Relative abundance (RPKM) of individual ODZ NOB groups ranged from zero in the upper, oxygenated surface waters to 6.85 RPKM (NOB3, 1.51% of total sequences) at 800 m in ETNP station PS3 (Fig. S3C). The combined RPKM of all ODZ NOB groups reached 15.37 RPKM (3.34% of the total sequences) in the ODZ of ETNP station 14 (Fig. 2, Fig. S3D). There was little difference in observed NOB communities between the ETNP and Arabian Sea OMZs, despite their large geographical distance, with NOB1, NOB2, and NOB3 dominating at ODZ depths. There were slightly higher relative abundances of NOB7, NOB8, and NOB9 in the Arabian Sea OMZ and slightly more NOB4 and NOB5 in the ETNP stations (Fig. S3). The distribution of individual ODZ NOB groups was similar between 2016 and 2018 in the ETNP, although NOB were more abundant in 2016.

ODZ NOB MAGs outnumbered any other identified NOB in all three major OMZs (Arabian Sea, ETNP, and ETSP) (Fig. 2, Fig. S3). In general, NOB MAG relative abundance was minimal at the surface and increased in the ODZ, with all individual ODZ NOB MAGs peaking in the 200 m below the oxic-anoxic interface, except for NOB3, whose maximum relative abundance was deeper near the lower oxycline (Fig. 2). The only OMZ station where ODZ NOB MAGs did not dominate throughout the entire profile was ETNP PS2, where SAG_1a was abundant at the upper and lower oxyclines (Fig. S3B). The relative abundance of cultured NOB and SAG_Nitrospirae was negligible (0.01–0.07 and 0.02–0.06 RPKM, respectively) at all depths in all OMZ stations, as was SAG_2a, except for PS2 850 m.

**Figure 3:**
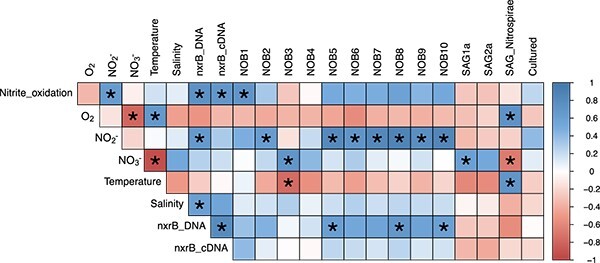
Pearson correlations comparing environmental variables and the RPKM of NOB groups from stations ETNP PS2, ETNP PS3, and ETNP PS6. A star (^*^) indicates a significant correlation (*P* <0.05). Colors represent Pearson correlation value. Environmental variables include: Nitrite oxidation rates (Nitrite_oxidation), concentration of O_2_, NO_2_^−^, NO_3_^−^, seawater temperature and salinity, the *nxrB* abundance based on DNA and cDNA, and the relative abundance (RPKM) of the each OMZ NOB group and each NOB SAG; cultured organisms were not included because some had zero relative abundance across all the included samples.

ODZ NOB MAGs were obtained from the same samples (ETNP stations PS1, PS2, PS3, and PS6) as previously published nitrite oxidation rates, which ranged from 0–57.5 nM N/day [3, 15]. The highest rates occurred where no oxygen was detected *in situ*, roughly within the first 100 m below the upper oxycline (Fig. 2). At the non-ODZ station, PS1, nitrite oxidation rates were more variable with depth, though the highest rate occurred at the bottom of the oxycline (Fig. 2A, Fig. S3A). The potential density surface of σt = 26.4 kg/m^3^ is taken as the demarcation between the oxycline and ODZ core [6]. At all stations, the highest rates of nitrite oxidation occurred at depths shallower than or very close to σt = 26.4 kg/m^3^ although nitrite oxidation rates significantly greater than zero were observed at σt > 26.4 (Fig. S4). Data on rates and metagenomes from the same samples allows a direct comparison of organisms and the processes attributed to them. At the depths of maximum nitrite oxidation, the nitrite-oxidizing community was dominated by NOB1, NOB2, and NOB3, with all other ODZ NOB present at lower abundances (Fig. 2, Fig. S3). Nitrite oxidation rates were significantly, positively correlated with the relative abundance of NOB1 (*r* = 0.72; Fig. 3, Fig. S5), and positively correlated with all other ODZ NOB relative abundance except for NOB3 and NOB4. There was a non-significant negative correlation between nitrite oxidation rates and SAG NOB abundances.

Using the new *nxrB* primers (see supplemental discussion 2, Fig. S7) for qPCR, *nxrB* gene abundance ranged from 1258 (±73, standard error) copies/mL in the surface waters of station PS3 to 301 326 (±4522) copies/mL in the ODZ of station 14 (Fig. 2; Fig. S3). The abundance of *nxrB* in cDNA was lower than in DNA, which is not surprising due to the rapid decay of RNA, from 4412 (±1000) copies/mL at 800 m at PS2 to 46 405 (±1661) copies/ml in the ODZ of PS3 (Fig. 2). *nxrB* expression levels may have been impacted by the sampling process despite filtering the samples as rapidly as possible and limiting oxygen exposure to the best of our abilities. *nxrB* abundance and relative abundance of NOB was higher in the 2016 ETNP stations (PS6 and 14) than in 2018 (PS2 and PS3). ETNP PS1, the station outside the OMZ, had the lowest abundance of *nxrB* genes and the lowest rates of nitrite oxidation (Fig. 2A).

Nitrite oxidation rates, *nxrB* abundance and expression, and ODZ NOB relative abundance, all obtained from the same samples, displayed similar depth profiles; all had maxima, often in the same samples, within 200 m below the oxic-anoxic interface where no oxygen was detected (Fig. 2A). *nxrB* abundance and expression were significantly (*P* < 0.05), positively correlated with each other (*r* = 0.80) and with nitrite oxidation rates (*r* = 0.75 and 0.70, respectively; Fig. 3). *nxrB* abundance and expression were positively correlated with ODZ NOB abundances, and negatively correlated with SAG NOB abundances (Fig. 3).

The position of sequences of expressed *nxrB* genes from stations PS1, PS2, PS3, and PS6 in the *nxrB* phylogenetic tree (Fig. S2) indicates that the active NOB were closely related to NOB1, NOB2, and SAG_1a. Therefore, the phylogeny of the cloned *nxrB* genes (see supplemental discussion 3) indicates that active NOB in ODZs are likely ODZ NOB MAGs, consistent with the metagenomic analysis.

### Genetic potential of oxygen-deficient zone nitrite-oxidizing bacteria

**Figure 4:**
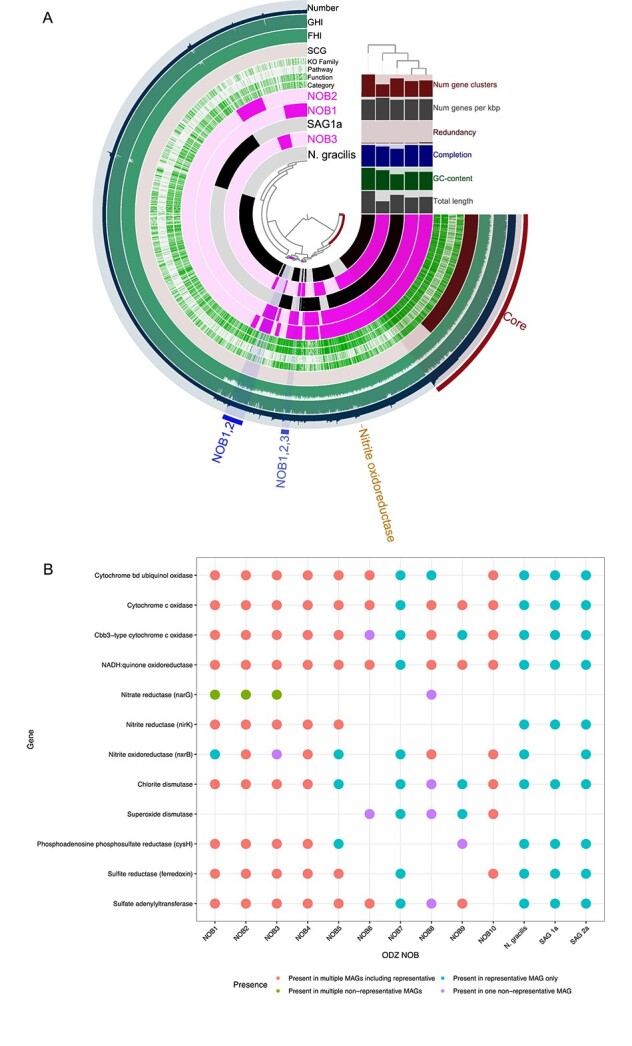
(A) Pangenomic analysis of gene clusters present in ODZ NOBs NOB1, NOB2, NOB3, as well as SAG1a (AG-538-K21, IMG genome 2681812871) and *N. gracilis* strain 3/211 (NCBI: SAMEA2272521) genomes. Gene clusters shared across all five genomes are labeled Core; gene clusters shared across NOB1 and NOB2 genomes are labeled NOB1,2 and across all three ODZ NOBs are labeled NOB1,2,3. The location of the nitrite oxidoreductase gene cluster is also indicated. Layers of the figure are, from top to bottom, the number of genes in the gene cluster (Number), the geometric homogeneity index (GHI), the functional homogeneity index (FHI), the presence of single copy gene (SCG) clusters, the KO family, the COG20 pathway (Pathway), the COG20 function (Function), the COG20 category (Category), the presence of a gene cluster in NOB2, NOB1, SAG1a, NOB3, and *N. gracilis.* For the KO family and the COG20 layers, known is shown in green and unknown is shown in white. Included in the bar graphs are the number of gene clusters, number of genes per kilobasepair, redundancy, completion, GC content, and total length of each genome. (B) The presence (dot present) or absence (no dot present) of genes of interest in each of the ODZ NOB groups, *N. gracilis*, SAG1a, and SAG2a (AG-538-L21, IMG genome 2681812872). Color of the dot represents the number of MAGs the gene was present in.

The pangenome analysis of the most abundant ODZ NOB (NOB1, NOB2, and NOB3), the most abundant NOB from the oxygenated ocean (SAG_1a; AG_538_K21), and the complete genome of the closest cultivated relative (*N. gracilis* strain 3/211) identified 4692 gene clusters. Although each genome had unique gene clusters (not shared with any other genome), the five genomes shared a core set of just over 900 gene clusters (Fig. 4A; Table S5), which included all single copy housekeeping genes, the major genes for carbon fixation through the rTCA cycle previously observed in the *N. gracilis* genome [25], chlorite dismutase, and genes for the cytochrome bc complex and the F_0_F_1_-type ATP synthase. Nitrite oxidoreductase was not present in the core set because it was missing from the NOB3 genome; *nxr* from the other 4 examined genomes fell into the same gene cluster (Fig. 4A) and a different NOB3 genome did contain *nxrB* (Fig. 4B).

The ODZ NOB genomes shared some gene clusters that were not present in either SAG_1a or *N. gracilis*. NOB1 and NOB2 shared a group of about 100 clusters and all three ODZ NOB shared about 40 clusters (Fig. 4A, Table S5). Just under half of the gene clusters shared by ODZ NOB and not present in SAG_1a or *N. gracilis* were uncharacterized and their function could not be determined. The remaining gene clusters included genes for transcription and translation regulation, stress responses, ion transport and uptake, and energy metabolism, including a cbb3-type cytochrome oxidase, an NADPH nitrite reductase, and an NADPH:quinone reductase.

All ODZ NOB groups, as well as *N. gracilis* and both SAGs, contained the genes for cytochrome c oxidase, the high affinity cbb3-type cytochrome c oxidase, NADH:quinone oxidoreductase and, except for NOB9, cytochrome bd ubiquinol oxidase (Fig. 4B); no examined NOB had the lower affinity aa3-type oxidase or cytochrome o ubiquinol oxidase. The only ODZ NOB lacking *nxrB* were NOB6 and NOB9. Nitrite reductase (*nirK*) was present in NOB1, 2, 3, 4, 5, *N. gracilis*, and both SAGs. Nitrate reductase was present only in NOB1, 2, 3, and 8, and urease was present in NOB1, 2, 3, 7, and 10 (Fig. 4B). Unlike *N. gracilis*, ODZ NOB6, 7, 8, 9, and 10 contained a superoxide dismutase. Genes involved in sulfur cycling, including phosphoadenosine phosphosulfate reductase, sulfate adenylyltransferase, and sulfite reductase were present in the majority of the examined NOB genomes (Fig. 4B); genes for sulfur oxidation (i.e. *soxB*) were only found in NOB6. Only NOB5 contained genes for fumarate reductase.

### Global biogeography of nitrite-oxidizing bacteria

**Figure 5:**
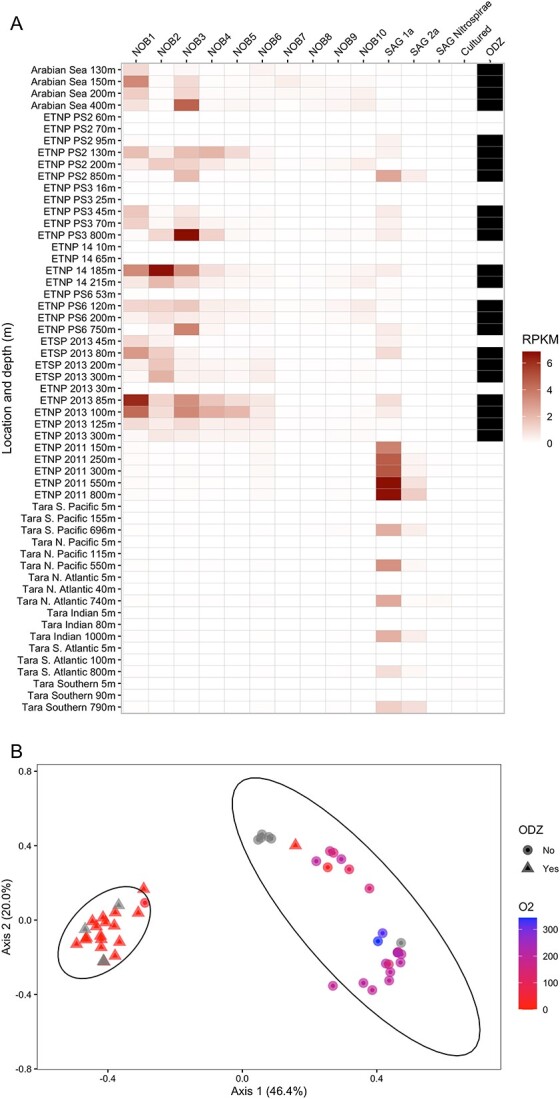
(A) Relative abundance (RPKM) of NOB in metagenomes from this study and previous studies (see Tables S1 and S4). ODZ indicates the sample location in the ODZ (black, <10 μM O_2_) or outside the ODZ (white, >10 μM O_2_). NOB and SAG groups are represented by their associated representative genome (see Tables S2 and S3); cultured NOB is the average RPKM of all considered cultured NOB. (B) A PCoA of the NOB communities, based on RPKM, in each sample. Shape represents whether the sample came from an ODZ (<10 μM O_2_) or not (>10 μM O_2_); color represents the O_2_ concentration. Gray was used when the exact O_2_ concentration was unknown. Data ellipses were calculated using a multivariate *t*-distribution.

The biogeography and relative abundance of the new ODZ NOB, previously reported NOB genomes and cultured representatives in 32 OMZ metagenomes (23 from this study, 9 previously published) and 23 previously published non-OMZ metagenomes representing major ocean biomes (Table S4), indicate clear niche separation between ODZ NOB and all other NOB. ODZ NOB MAGs were found in previously published OMZ metagenomes from the ETSP [26] and the ETNP (ETNP_2013) [57], with depth profiles similar to those reported here (Fig. 5A). Two ODZ NOB groups found in this study, NOB1 and NOB2, were the same “species” as NOB MAGs previously found in the ETSP OMZ; the relative abundance depth profile of NOB1 and NOB2 in the same ETSP metagenomes closely matched those previously reported (Fig. S3F) [14, 26]. All ODZ NOB groups were observed in very low abundances in metagenomes from just outside of the ETNP OMZ (ETNP_2011; Fig. 2C, 5A) [21] and were not detected in the oxygenated open ocean of the major ocean basins (Tara; Fig. 5A) [58].

The cultured NOB were negligible (0–0.09 RPKM) in all environmental samples (Fig. 5A). SAGs isolated from oxygenated mesopelagic zones [19] were present at the edges of the ODZ (upper and lower oxycline) in OMZ metagenome samples and were rarely detected in the ODZ (Fig. 5A, Fig. 2). SAG_1a was found in higher abundance in the oxygenated mesopelagic samples from all major ocean basins and in the entire depth profile from the non-OMZ ETNP metagenome (ETNP_2011) where it reached 6.81 RPKM, the equivalent of 1.73% of all sequenced reads, at 550 m (Fig. 2C). In contrast, the highest relative abundance of SAG_1a in an OMZ sample was 2.59 RPKM (Fig. 5A). SAG_2a was present in the same samples as SAG_1a, but at lower abundance; SAG_Nitrospirae was present in only a few metagenomes at very low abundances (Fig. 5A).

A PCoA based on relative abundance of all NOB genomes, MAGs, and SAGs separated the NOB communities into two major groups, one at higher oxygen levels (including upper and lower oxycline samples), and the second from ODZs (Fig. 5B). The ODZ group of communities included samples from this study and from the ODZ of the ETNP_2013 and ETSP_2013 [26, 57]; the other group included all samples from the oxygenated open ocean [58] as well as samples from OMZ upper and lower oxyclines and the outer boundary of an OMZ (ETNP_2011) [21]. A smaller component of the variability among communities was associated with depth, with shallower samples separating from deeper mesopelagic samples. This is particularly evident in the oxygenated samples (Fig. 5B), which separated between surface and mesopelagic. The separation of NOB communities by environmental (oxygen and depth) factors rather than by station or geographic region supports the idea that NOB present in ODZs are uniquely adapted to that environment, regardless of the geographic location of the OMZ.

## Discussion

### Oxygen-deficient zones harbor a distinct community of nitrite-oxidizing bacteria

Thirty-one NOB MAGs belonging to 10 different “species” were identified from OMZ metagenomes. The most abundant NOB belonged to the class *Nitrospinia*, consistent with the dominance of *Nitrospina* in previous reports from ETNP and global oceans [7, 19, 21, 23]. The ODZ NOB groups are distinct from cultured NOB and NOB previously identified to be important in the open ocean [19]. Several Clade 1 *Nitrospina*-like organisms, including SAG_1a (AG-538-K21), have been grouped into a suggested genus, *Candidatus* Nitromaritima, which was phylogenetically distinct from *N. gracilis* and abundant in the ETSP OMZ and the Red Sea [59]. The high diversity of NOB present in OMZs and their distant phylogenetic relationship to other NOB suggest that nitrite oxidizers are more diverse than previously recognized, supporting the conclusions of a recent analysis of the *Nitrospinota* phylum [20]. ODZ NOB were only closely related to other NOB from OMZ regions, not to NOB from oxic or seasonally anoxic regions, implying that these ODZ NOB are adapted specifically to permanently oxygen-depleted environments.

The clear contrast between distribution of NOB at OMZ vs. open ocean stations indicates strong niche separation in NOB communities with ODZ NOB MAGs specializing in ODZ environments. The pangenomic and genome-based analyses identified a core genetic component shared between ODZ and non-ODZ NOB with a smaller subset of genes unique to each group and to individual species. Based on the shared similarity in core metabolic functions, both ODZ and non-ODZ NOB are likely to be able to function as aerobes. Some genes, like the high-affinity cbb3-type cytochrome c oxidase [60], likely support NOB growth in low-oxygen waters; the existence of these genes in both ODZ NOB and NOB from oxic systems shows that the evolutionary advantage of these microaerophilic adaptations exists outside of the ODZ. The pangenome comparison revealed cbb3-type cytochrome c oxidase and other energy metabolism genes belonged to different gene clusters in the ODZ NOB and SAG_1a and *N. gracilis*, potentially implying a gene-level variation in these key enzymes, though more research into these and other shared genes is needed to determine if these gene-level variations result in functional changes. Although there are no obvious gene clusters that enable the ODZ MAGs to survive and outcompete other NOBs in the ODZs, the presence of gene clusters unique to ODZ MAGs supports their genetic distinction from other NOB and may in the future provide clues to the specific adaptations of ODZ NOBs, especially as currently unknown proteins are characterized.

The genome analysis of ODZ MAGs supports many of the findings from the recent investigation into the *Nitrospinota* phylum including the presence of *nirK* and urease in *Nitrospinia* MAGs, the presence of *soxB* in class UBA7883 (NOB6) and a potential role of *Nitrospinota* in sulfur cycling, and the conservation of autotrophic growth by the rTCA cycle across all *Nitrospinota* genomes [20]. Recent research has also suggested that the class *Nitrospinia* are the most abundant and active nitrite oxidizers in the ocean and that other *Nitrospinota* may play other roles [20], though more research needs to be done to evaluate this hypothesis. The *Nitrospinia* ODZ MAGs (i.e. NOB 1, 2, and 3) were the most dominant ODZ NOB and NOB1 and 2 had the closest relationship with nitrite oxidation rates in the OMZs. Although these genome-based analyses can provide valuable information on the potential roles of these organisms, further research is required to understand the controls on these genomes in the marine environments and which genes are tied to their survival in the ODZ niche. This work provides the first step in better understanding the ODZ NOB by exploring their global biogeography and linking their presence to activity in the ODZ.

### Oxygen-deficient zone nitrite-oxidizing bacteria are responsible for nitrite oxidation in oxygen-depleted waters

The highest rates of nitrite oxidation in OMZs are consistently reported in oxygen-depleted waters, though experimental oxygen manipulation experiments document variable responses of nitrite oxidation to oxygen [1, 3, 6, 7, 10, 61, 62]. The rates represented in this paper are no exception, with the highest nitrite oxidation rate occurring in the ODZ in all stations, generally coinciding with the highest abundance and expression of *nxrB*. The strong correlation between nitrite oxidation and *nxrB* abundance and expression, even considering that the *nxrB* abundance may overestimate the cell number because *nxrB* often occurs in two copies in *Nitrospina* genomes [25], supports the idea that nitrite oxidation is actively being performed by NOB in the ODZ. The high identity between *nxrB* from ODZ NOB and *nxrB* cloned from cDNA at the nitrite oxidation peak (see supplemental discussion 3) shows that the NOB performing the nitrite oxidation are the newly identified ODZ NOB. Furthermore, the strong correlation between the relative abundance of ODZ NOB, especially NOB1, and nitrite oxidation rates adds additional support for identity of the active members of the NOB community. Overall, the biogeochemical and molecular analyses from the same samples indicate that the ODZ NOB MAGs represent the primary organisms responsible for nitrite oxidation in ODZ waters; the NOB SAGs previously identified to be critical for marine nitrite oxidation [19] are only prevalent in oxygenated waters.

Many mechanisms have previously been proposed to explain apparently anaerobic nitrite oxidation, i.e. nitrite oxidation that occurs in oxygen-depleted waters, including: (i) canonical aerobic nitrite oxidation supported by sporadic intrusions of oxygen [63], (ii) use of an alternative electron acceptor such as iodate [10], (iii) nitrite dismutation [64], (iv) isotopic equilibration catalyzed by the reversible NXR protein, resulting in isotopic enrichment of the nitrate pool without net production of nitrate [65–67], and (v) cryptic oxygen cycling between *Prochlorococcus* and NOB at the deep chlorophyll maximum [67]. A full discussion of these suggested explanations and their relevance to the observed nitrite oxidation in OMZs has recently been published [8].

More recent modeling work has suggested that infrequent oxygen intrusions could support high rates of aerobic nitrite oxidation [68]. Although no oxygen intrusions were detected in STOX sensor profiles during sample collection, the highest ODZ NOB abundances, nitrite oxidation rates, and gene expression abundance occurred at depths shallower than or close to σt = 26.4 kg/m^3^. At depths shallower than σt = 26.4, intrusions of oxygen are considered possible, if infrequent; at deeper depths, significant impact of such intrusions is considered negligible [4]. This is also the depth where, based on the stoichiometry of organic matter remineralization, >50% of the nitrite produced in the OMZ is reoxidized [18].

The exact mechanism behind nitrite oxidation in the absence of detectable oxygen needs further study, but the presence of microaerobic core metabolism in ODZ NOB suggests that infrequent intrusions of oxygen-bearing water [68] are the most likely explanation. Anaerobic metabolism cannot be ruled out, however, as further analysis of uncharacterized genes present in ODZ NOB MAGs may reveal unknown functions. The ODZ NOB are responsible for nitrite oxidation in the apparent absence of oxygen and therefore for the retention of fixed nitrogen in OMZs. Further exploration of their genomes may reveal the adaptations that make them unique from all other NOB and allow them to exploit and dominate this niche.

## Supplementary Material

Fortin_et_al_supplemental_materials_ISMErevision_edited_wrae160

Fortin_et_al_Supplemental_Tables_ISMErevision_wrae160

## Data and materials availability

Metagenomic data and MAGs used in this study are available at JGI (Proposal ID 503962) and NCBI (BioProject PRJNA1003508). More information on the ETNP 2018 samples can be found in BCO-DMO project 741 018. Code used in this analysis can be found at https://github.com/sgfortin/ODZ-NOB.
