# Supplementary material for: Nitrite-oxidizing bacteria adapted to low-oxygen conditions dominate nitrite oxidation in marine oxygen minimum zones
Source: ISME J. 2024 Aug 14;18(1):wrae160. doi: 10.1093/ismejo/wrae160 (PMC11373643; doi:10.1093/ismejo/wrae160)
Supplement: Fortin_et_al_supplemental_materials_ISMErevision_edited_wrae160 [file fortin_et_al_supplemental_materials_ismerevision_edited_wrae160.docx]

Supporting information for

**Low oxygen specialist nitrite oxidizing bacteria dominate nitrite oxidation in oxygen minimum zones**

Samantha G. Fortin, Xin Sun, Amal Jayakumar, Bess B. Ward

This file includes:

Supplemental Methods

Supplemental Discussion 1-3

Supplemental Figures S1-S7

In additional excel file:

Supplemental Tables S1-S6

**Supplemental Methods**

*Additional Metagenomic Samples: Incubation, Sampling, and Sample Processing*

Two depths were sampled at each station representing the shallow chlorophyll maximum and the deep chlorophyll maximum using 30-L Niskin bottles on a Seabird CTD rosette system before dawn. The Niskin bottles were plumbed with CO_2_ (in the headspace, not sparging the water) while water was being transferred to the incubation bottles in order to minimize air contamination. Water was transferred directly to 4-L clear polycarbonate bottles wrapped in black plastic, filled to overflowing, and capped without bubbles or a headspace. Samples (TF) were incubated until late afternoon (Table S1) wrapped in opaque plastic in deck incubators at about 0.1% of surface light intensity and at surface seawater temperature of 27^o^C, then filtered onto 0.22µm Sterivex capsules, frozen in liquid nitrogen, and stored at -80^o^C until extraction using the All-Prep DNA/RNA Mini Kit. The quality of extracted DNA was verified by the 2100 Bioanalyzer System (Agilent, Santa Clara, CA). Randomly amplified DNA fragments were pair-end sequenced with Illumina HiSeq (Illumina, San Diego, CA) (180nt paired end). Quality check, random amplification, and sequencing were conducted by the Genomics Core Facility of Lewis-Sigler Institute for Integrative Genomics at Princeton University.

*Primer Design and Development*

The beta subunit of the nitrite oxidoreductase gene (*nxrB*), the gene commonly used for NOB phylogeny and identification [1], was identified in the NOB MAGs and in contigs from a subset of the ETNP samples using blast+ [2] and HMMER [3] with search templates based on previously identified *nxrB* gene sequences [4] and the *nxrB* model from the KBase HMMs of Environmental Bioelement application [5]. The resulting sequences were confirmed as *nxrB* by comparing to the NCBI GenBank database. An alignment was made of confirmed *nxrB* genes using MEGA v11 [6] and used to search *in silico* for previously published *nxrB* primer sequences. Sequences matching existing primers were only found in a few OMZ *nxrB* sequences, therefore new primers were developed to target Nitrospinae *nxrB* genes from OMZs: nxrBomz1F (5’ GAGTAYATGTGGTGGAAYAAYGT) and nxrBomz1R (5’ CRTCCTCYTGGCGYTTGTA). The new primer set was checked for specificity using a blastn search against the NCBI GenBank database and was further confirmed by gel electrophoresis and the sequencing of clonally amplified fragments.

*PCR Conditions, Creation of Standard, and Cloning*

PCR conditions for amplification with primers nxrBomz1F and nxrBomz1R: 5μL of GoTaq buffer, 0.125μL of GoTaq, 0.5μL of dNTPs, 1μL of each primer (10pmol/μL), and 2μL of DNA, with the rest of the 25μL reaction consisting of water. The PCR program was: 95^o^C for 3min followed by 35 cycles of 95^o^C for 30s, 50^o^C for 40s, and 72^o^C for 1min, with a final elongation step of 72^o^C for 7min. Triplicate reactions were combined and purified using the Qiagen QIAQuick Gel Extraction kit and manufacturer protocols.

*qPCR Optimization*

The new primer set was used to amplify *nxrB* genes from a separate (not metagenomically sequenced) ETNP OMZ sample from 70m depth. The purified gene product from triplicate reactions was then inserted into a pGEM-T Easy Vector (Promega) and cloned into *E. coli* cells using established protocols. Cloned inserts were sequenced and confirmed to be *nxrB* with high identity to *nxrB* identified in the metagenomic samples. One cloned *nxrB* amplicon was used as a standard for qPCR.

The standard curve and 3 representative samples were used to optimize the qPCR reaction over a range of annealing temperatures (50 to 58^o^C), primer additions (0.5 to 2μL of each primer), and template DNA additions (1 to 2μL). The optimized reaction included 6.25μL of QuantiTect master mix (Qiagen), 1μL of each primer (10pM; nxrBomz1F/R), and 1μL of template DNA or cDNA (ranging between 0.02 and 35ng/μL), with the rest of the 12μL reaction made up of water. The optimized qPCR protocol was as follows: 95^o^C for 10 min, followed by 40 cycles of 95^o^C for 30s, 52^o^C for 40s, and 72^o^C for 30s, followed by a melt curve to check for specificity of the reaction.

Based on the standard curve, created through a serial dilution of an *nxrB* cloned from an ETNP sample, the efficiency of the qPCR ranged between 46.6 and 54.7%. The efficiency of each individual reaction was also calculated based on the amplification curve [7] using the LinRegPCR program [8]. Using this method of efficiency calculation, *nxrB* DNA standards and samples had an efficiency of 86.9 and 85.2%, respectively. Due to the similar efficiency of standards and samples in individual reactions, the gene copy numbers determined from the standard curve are likely to accurately represent the true copy numbers in the environmental samples, despite the low standard curve-based efficiency. The primers used in the qPCR had several degeneracies, three each in the forward and reverse, in order to cover the wide range of *nxrB* found in the samples from three oxygen minimum zones which likely lowered the efficiency of the reaction.

**Supplemental Discussion 1: Characteristics of OMZ stations**

Samples were collected from the ETNP in two different years, and from the Arabian Sea (Figure S1). Each OMZ station had a sharp oxycline, an anoxic OMZ core (also referred to as the oxygen depleted zone, ODZ), and a primary and secondary nitrite maximum. Of the three 2018 ETNP stations, PS2 and PS3 were in the OMZ and had large secondary nitrite maxima, while PS1 bordered the OMZ and, though it had an ODZ, had only one nitrite maximum located in the oxycline making it a non-OMZ station (Figure S6). Station PS3 was a coastal station and had a shallower oxycline depth than the two offshore stations. Two ETNP OMZ stations, PS6 and 14, were sampled in 2016. PS6 was a coastal station with a shallower oxycline depth than 14 (Figure S6). Only one station was sampled for metagenomic work in the Arabian Sea OMZ in 2007; it showed the characteristic oxygen depleted zone and larger secondary nitrite peak (Figure S6).

**Supplemental Discussion 2: Quantifying NOB organisms**

Relative abundance, even normalized by RPKM, can be influenced by a number of factors in OMZs as microbial diversity is influenced by depth and oxygen concentrations [9]. To account for any potential inaccuracies in the abundance of MAGs, the actual abundance of *nxrB* in both DNA and cDNA was determined using qPCR with primers that captured *nxrB* genes found in the ODZ NOB and the Clade 1a Nitrospinae. While *nxrB* is often found in two copies in *Nitrospina* genomes [10], the DNA based qPCR data still provides a more accurate count of how many *nxrB* genes, and therefore how many NOB, are present throughout the depth profile. The need for both relative and quantitative measurements can be seen in the conflicting observations of NOB3; the observed increase in RPKM of NOB3 at depth was not reflected in an increase in the DNA based abundance of *nxrB*. Therefore, it is likely the increase in NOB3 at depth indicates a shift in its importance in the entire community rather than an increase in the number of NOB3 cells at depth. Despite the discrepancy between NOB3 relative abundance and qPCR abundances, there is a significant positive linear relationship between the DNA based qPCR abundance of *nxrB* and the relative abundance of all NOB (i.e., ODZ NOB, SAG NOB, and cultured NOB combined), showing that the metagenome and qPCR analyses support each other (Figure S7).

**Supplemental Discussion 3: Phylogenetic relationship of expressed *nxrB* genes**

The *nxrB* tree included: sequences cloned from the cDNA of the depth with the highest nitrite oxidation rate at stations ETNP PS1, ETNP PS2, ETNP PS3, and ETNP PS6, sequences obtained from ETNP PS2 130m and ETNP PS3 70m metagenomes, and sequences from OMZ MAGs, SAGs, and cultured organisms (Figure S2). Most branches in the tree were very short, making identification of NOB based on their *nxrB* genes difficult. However, one clade of the tree clearly grouped with *nxrB* genes from multiple NOB2 MAGs (yellow clade Figure S2) while a second grouped closely with one of the *nxrB* genes from NOB1 (green clade Figure S2). However, many of the sequences fell on a branch of the tree that included a *nxrB* gene from NOB1 and *nxrB* genes from SAG 1a (blue clade Figure S2). Two more large groups (Collapsed clades N86 and N42 Figure S2) contained *nxrB* genes only from clones and the metagenomes; this group is likely represented in the OMZ MAGs but, since not all MAGs contained *nxrB,* could not be identified at this time. While this suggests that *nxrB* may not be the ideal gene to separate the Clade 1a Nitrospinae at the species level, an endeavor that is likely complicated by NOB containing multiple copies of the *nxrB* gene, the cloned cDNA *nxrB* do indicate that the corresponding metagenomes are accurately representing the NOB community and its dominance by ODZ NOBs. Furthermore, since the cDNA was used for cloning, these NOB communities are actively producing RNA for *nxrB* at depths where nitrite oxidation is also present.

**References**

1. Pester M, Maixner F, Berry D, Rattei T, Koch H, Lücker S, et al. NxrB encoding the beta subunit of nitrite oxidoreductase as functional and phylogenetic marker for nitrite-oxidizing Nitrospira. *Environ Microbiol* 2014; **16**: 3055–3071. https://doi.org/10.1111/1462-2920.12300.

2. Camacho C, Coulouris G, Avagyan V, Ma N, Papadopoulos J, Bealer K, et al. BLAST+: Architecture and applications. *BMC Bioinformatics* 2009; **10**: 421. https://doi.org/10.1186/1471-2105-10-421.

3. Eddy SR. Accelerated profile HMM searches. *PLoS Comput Biol* 2011; **7**: e1002195. https://doi.org/10.1371/journal.pcbi.1002195.

4. Sun X, Kop LFM, Lau MCY, Frank J, Jayakumar A, Lücker S, et al. Uncultured Nitrospina-like species are major nitrite oxidizing bacteria in oxygen minimum zones. *ISME J* 2019; **13**: 2391–2402. https://doi.org/10.1038/s41396-019-0443-7.

5. Anantharaman K, Brown CT, Hug LA, Sharon I, Castelle CJ, Probst AJ, et al. Thousands of microbial genomes shed light on interconnected biogeochemical processes in an aquifer system. *Nat Commun* 2016; **7**: 13219. https://doi.org/10.1038/ncomms13219.

6. Tamura K, Peterson D, Peterson N, Stecher G, Nei M, Kumar S. MEGA5: Molecular evolutionary genetics analysis using maximum likelihood, evolutionary distance, and maximum parsimony methods. *Mol Biol Evol* 2011; **28**: 2731–2739. https://doi.org/10.1093/molbev/msr121.

7. Ruijter JM, Ramakers C, Hoogaars WMH, Karlen Y, Bakker O, van den hoff MJB, et al. Amplification efficiency: Linking baseline and bias in the analysis of quantitative PCR data. *Nucleic Acids Res* 2009; **37**: e45. https://doi.org/10.1093/nar/gkp045.

8. Untergasser A, Ruijter JM, Benes V, van den Hoff MJB. Web-based LinRegPCR: application for the visualization and analysis of (RT)-qPCR amplification and melting data. *BMC Bioinformatics* 2021; **22**: 398. https://doi.org/10.1186/s12859-021-04306-1.

9. Ganesh S, Parris DJ, Delong EF, Stewart FJ. Metagenomic analysis of size-fractionated picoplankton in a marine oxygen minimum zone. *ISME J* 2014; **8**: 187–211. https://doi.org/10.1038/ismej.2013.144.

10. Lücker S, Nowka B, Rattei T, Spieck E, Daims H. The genome of Nitrospina gracilis illuminates the metabolism and evolution of the major marine nitrite oxidizer. *Front Microbiol* 2013; **4**: 00027. https://doi.org/10.3389/fmicb.2013.00027.


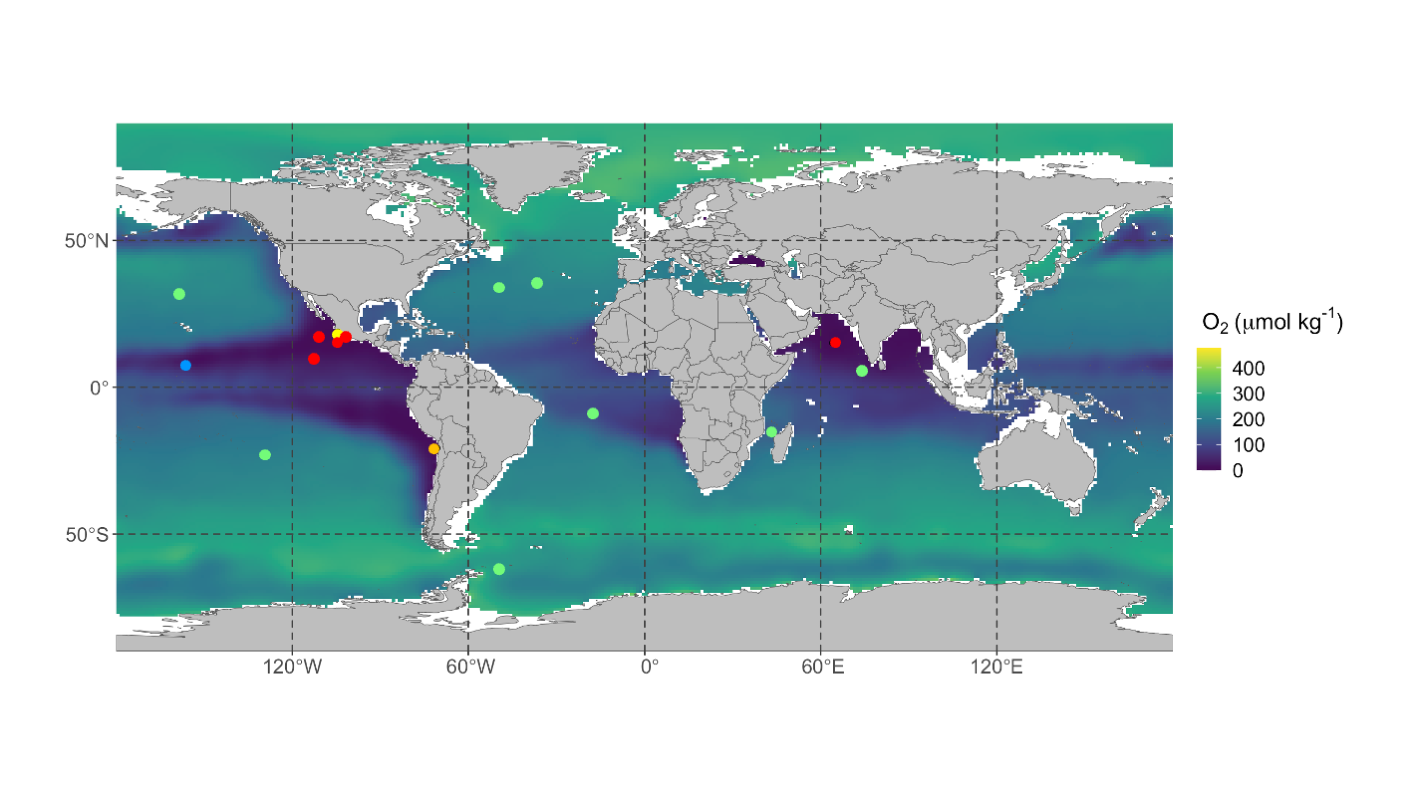
**Figure S1.** A map of all locations with metagenomic samples used in this study overlying the annual mean O_2_ concentration (from 1955 to 2017, World Ocean Atlas) at 200m below the surface. Red indicates OMZ samples from this study where new NOB MAGs originated. The remaining colors indicate the location of previously published metagenome samples: oxygenated open ocean (green; Tara Oceans), ETSP 2013 OMZ (orange), ETNP 2013 OMZ (yellow), and ETNP 2011 bordering the OMZ (blue). More details on the metagenomic samples can be found in Tables S1 and S4.

**Figure S2.** Phylogenetic tree of *nxrB* genes from NOB ODZ MAGs (purple), cultured NOB genomes (pink), Nitrospinae Clade 1a SAGs (red), cDNA clones (bold), and metagenome sequences. Blue clade represents sequences resembling NOB1 and SAG 1a, green clade represents sequences resembling NOB1, and yellow clade represents sequences resembling NOB2. Collapsed clade N86 includes *nxrB* sequences from: PS1 100m cDNA clones (n=38), PS6 120m cDNA clones (24), PS2 200m cDNA clones (7), PS2 130m metagenome (6), PS3 70m metagenome (6), and PS3 45m cDNA clones (4). Collapsed clade N42 includes *nxrB* sequences from: PS2 200m cDNA clones (17) and PS3 45m cDNA clones (3). Scale bar represents a branch length of 10.

**
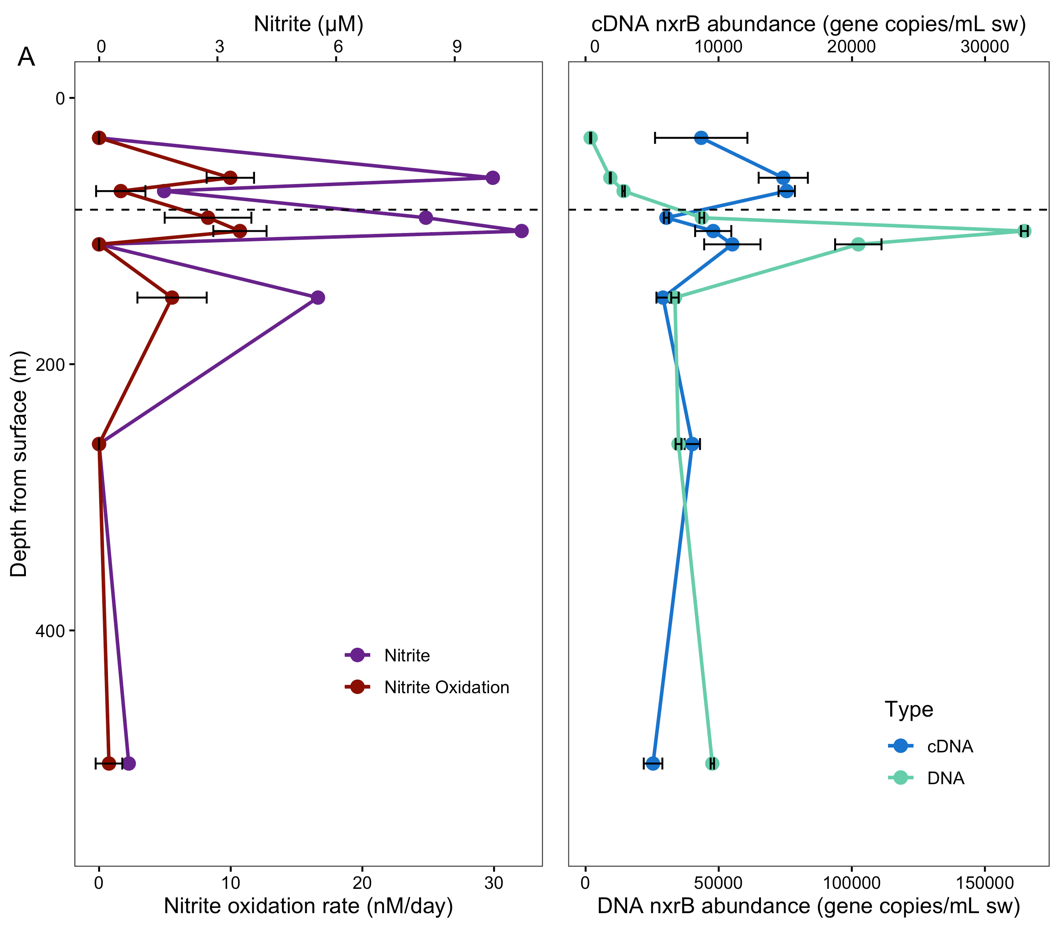
**

**
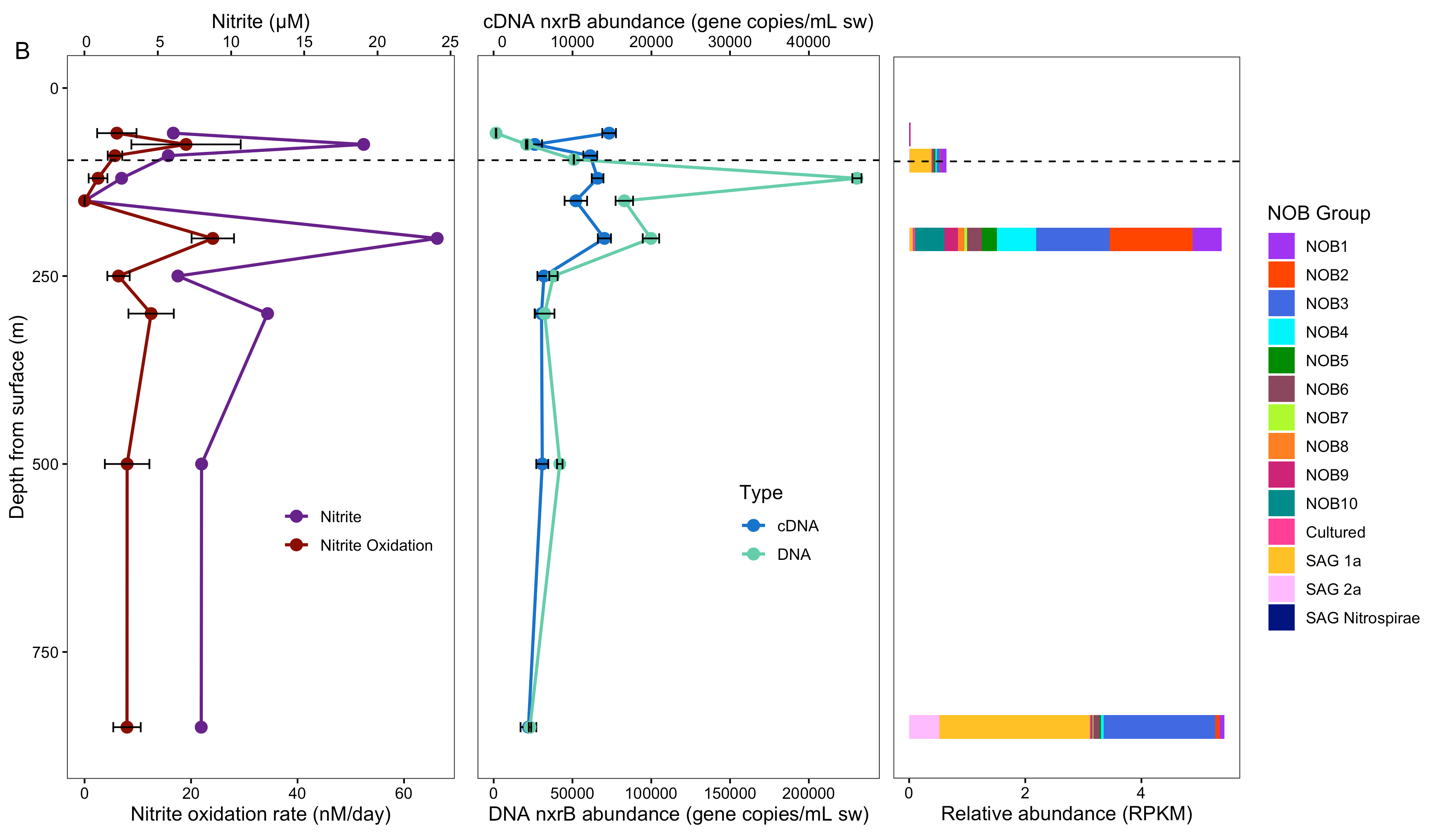
**

**
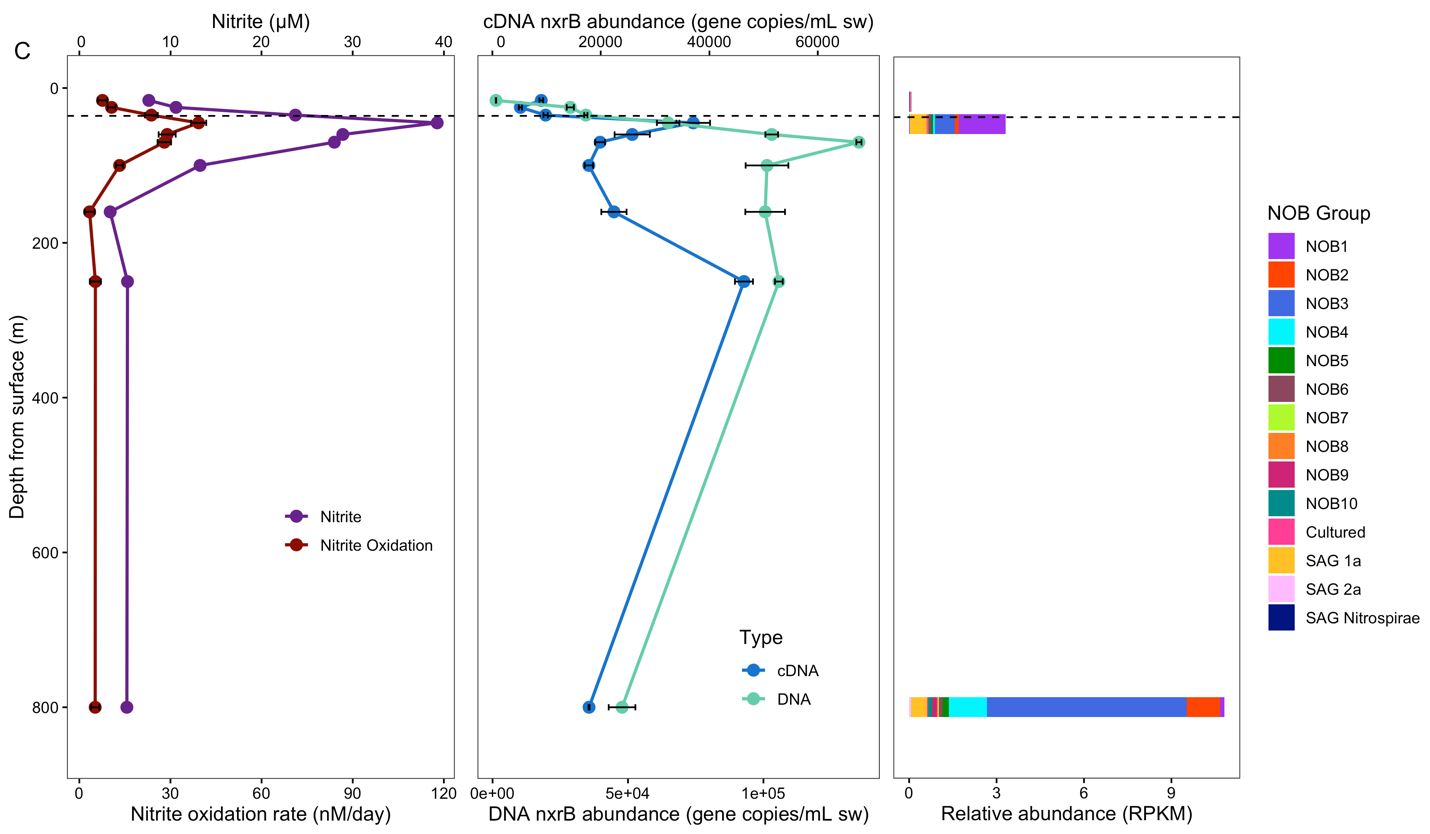
**

**
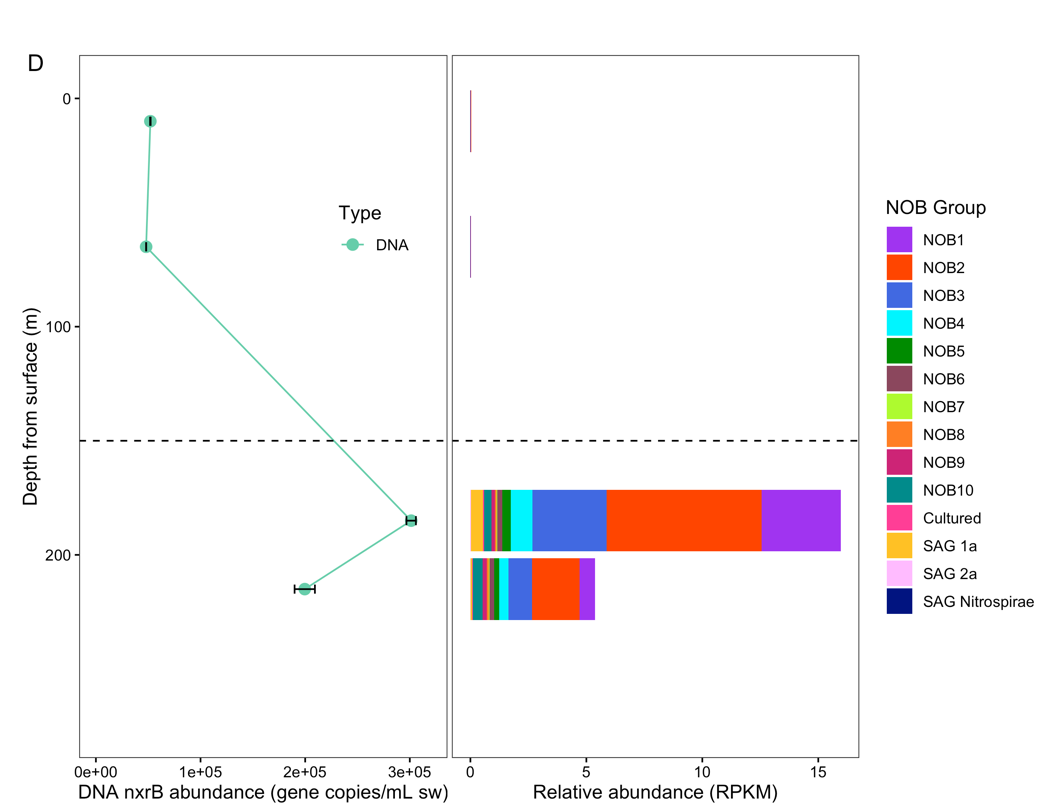
**

**
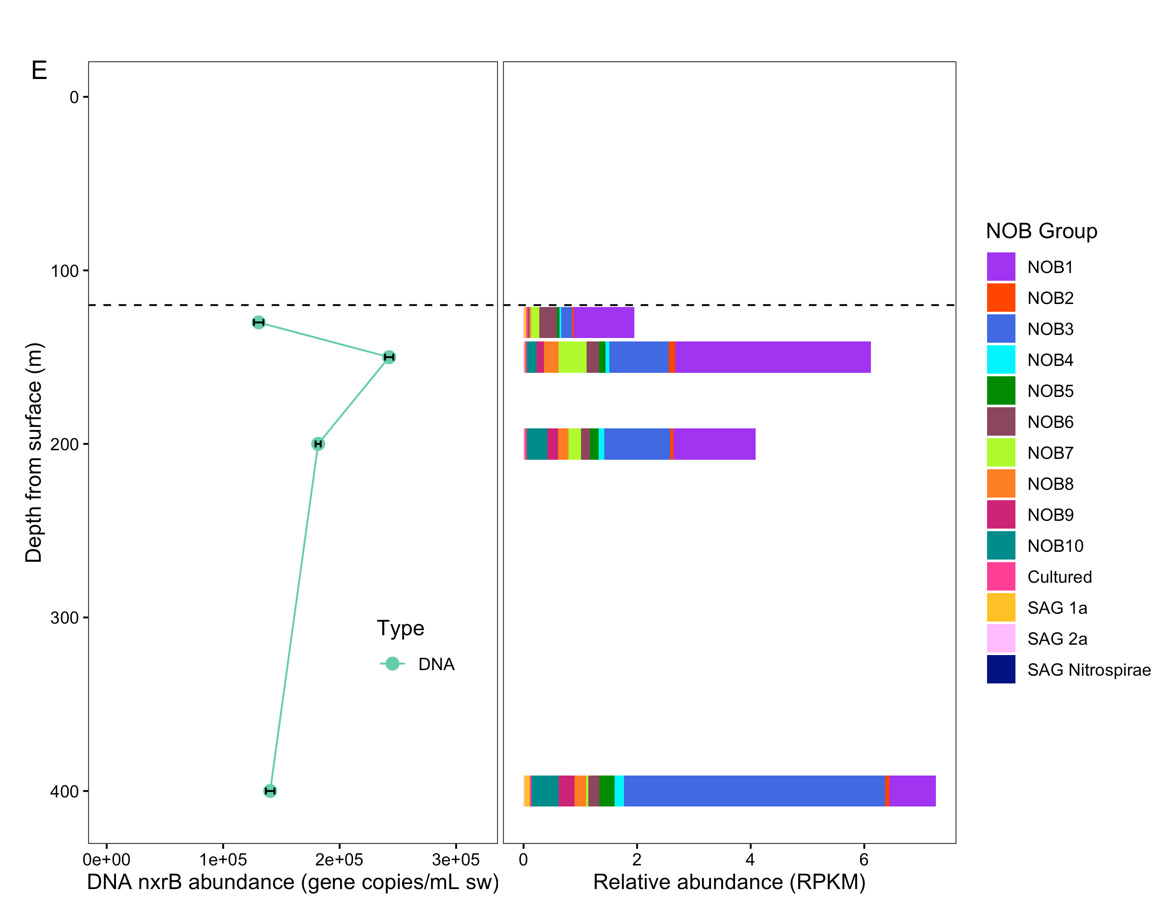
**

**
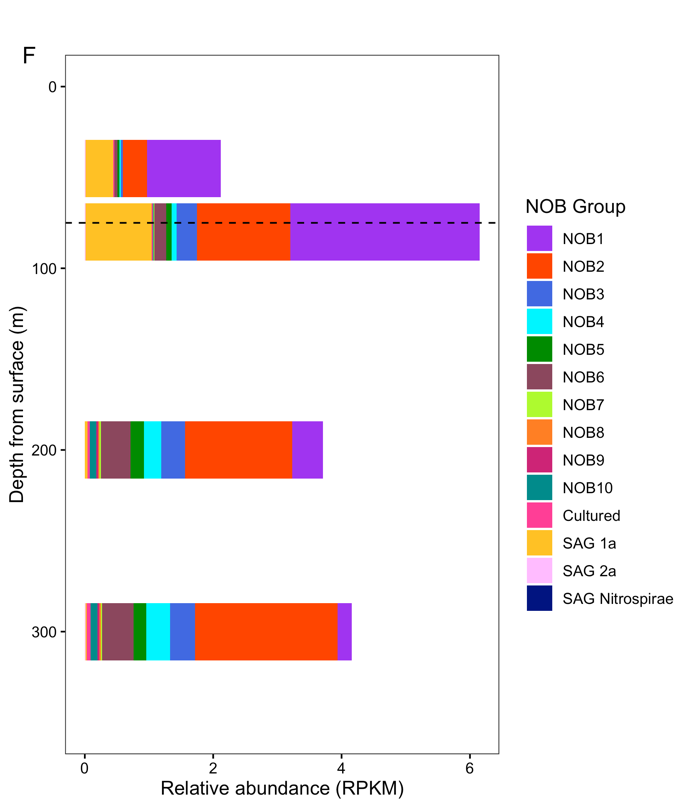
**

**Figure S3.** The nitrite oxidation rate (nM/day), DNA and cDNA based *nxrB* gene abundance (gene copies/mL seawater), and the abundance (RPKM) of NOB groups in station **(A)** ETNP PS1, **(B)** ETNP PS2, **(C)** ETNP PS3, **(D)** ETNP 14, **(E)** Arabian Sea, **(F)** ETSP 2013, plotted against the depth (m) below the surface. Error bars represent standard error; nitrite oxidation error bars are from biological replicates, *nxrB* abundance error bars are from technical replicates.

**Figure S4.** A scatter plot of density (σt, kg/m^3^) versus nitrite oxidation rates (nM/day) at stations ETNP PS1, PS2, PS3, and PS6. The black line represents a σt of 26.4.

**Figure S5.** Pearson correlations comparing environmental variables and the RPKM of NOB groups from Stations ETNP PS2, ETNP PS3, and ETNP PS6. A star (*) indicates a significant correlation (p<0.05). Colors represent Pearson correlation value. Environmental variables include: nitrite oxidation rates (Nitrite_oxidation), concentration of O_2_, NO_2_, NO_3_, seawater temperature and salinity, the *nxrB* abundance based on DNA and cDNA, and the relative abundance (RPKM) of the each ODZ NOB group and each NOB SAG; cultured organisms were not included because most had zero relative abundance across all the included samples. This figure shows the same data as Figure 3 for the first 8 rows.

**Figure S6.** Depth profiles of OMZ stations representing the concentration of nitrite (green), oxygen (pink), and PAR or fluorescence (purple). ETNP PS1, PS2, and PS3 had PAR measurements (microEinsteins/m^2^); ETNP 14 and ETNP PS6 had fluorescence (μg/L) measurements; Arabian Sea had neither PAR or fluorescence measurements.

**Figure S7.** DNA based *nxrB* abundance (gene copies/mL seawater) determined by qPCR compared to relative abundance (RPKM) of all NOB including ODZ NOB, SAG NOB, and cultured NOB. Line represents the linear regression (obtained using ggplot2 lm setting in geom_smooth function) with the adjusted R^2^ and p-value of the linear regression reported in the top left corner.
